# Electro-Physiology and Electro-Therapeutics

**Published:** 1861-03

**Authors:** 


Electro-Physiology and Electro-Therapeutics; showing the best methods for the Medical Uses of Electricity. By Alfred C. Garnett, M. D., &c. Second Edition. Boston: Ticknor & Fields, 1861.

This is a large and elegant volume of 716 pages octavo.—The first edition was pretty freely noticed and strongly recommended, in the pages of the Examiner only a few months since. Hence we will only add in reference to the second edition, that it contains several pages of additional matter, and several new illustrations. Both students and practitioners will find it a work of much intrinsic value. Price, $3. For sale at the extensive book-store of S. C. Griggs & Co., of this city.

